# Antifungal activity of probiotic strain *Lactiplantibacillus plantarum* MYSN7 against *Trichophyton tonsurans*

**DOI:** 10.3389/fmicb.2023.1192449

**Published:** 2023-06-14

**Authors:** P. R. Vanitha, Rakesh Somashekaraiah, S. Divyashree, Indranil Pan, M. Y. Sreenivasa

**Affiliations:** ^1^Department of Studies in Microbiology, University of Mysore, Mysuru, India; ^2^Maharani's Science College for Women, Mysuru, India; ^3^Department of Biosciences, School of Biosciences and Technology, Vellore Institute of Technology, Vellore, India

**Keywords:** *L plantarum*, probiotic, *Trichophyton tonsurans*, antifungal activity, lactic acid bacteria

## Abstract

The primary objective of this study was to assess the probiotic attributes and antifungal activity of lactic acid bacteria (LAB) against the fungus, *Trichophyton tonsurans*. Among the 20 isolates screened for their antifungal attributes, isolate MYSN7 showed strong antifungal activity and was selected for further analysis. The isolate MYSN7 exhibited potential probiotic characteristics, having 75 and 70% survival percentages in pH3 and pH2, respectively, 68.73% tolerance to bile, a moderate cell surface hydrophobicity of 48.87%, and an auto-aggregation percentage of 80.62%. The cell-free supernatant (CFS) of MYSN7 also showed effective antibacterial activity against common pathogens. Furthermore, the isolate MYSN7 was identified as *Lactiplantibacillus plantarum* by 16S rRNA sequencing. Both *L. plantarum* MYSN7 and its CFS exhibited significant anti-*Trichophyton* activity in which the biomass of the fungal pathogen was negligible after 14 days of incubation with the active cells of probiotic culture (10^6^ CFU/ml) and at 6% concentration of the CFS. In addition, the CFS inhibited the germination of conidia even after 72 h of incubation. The minimum inhibitory concentration of the lyophilized crude extract of the CFS was observed to be 8 mg/ml. Preliminary characterization of the CFS showed that the active component would be organic acids in nature responsible for antifungal activity. Organic acid profiling of the CFS using LC-MS revealed that it was a mixture of 11 different acids, and among these, succinic acid (9,793.60 μg/ml) and lactic acid (2,077.86 μg/ml) were predominant. Additionally, a scanning electron microscopic study revealed that CFS disrupted fungal hyphal structure significantly, which showed scanty branching and bulged terminus. The study indicates the potential of *L. plantarum* MYSN7 and its CFS to control the growth of *T. tonsurans*. Furthermore, *in vivo* studies need to be conducted to explore its possible applications on skin infections.

## Introduction

Dermatophytes are a group of pathogenic fungi that primarily cause superficial infections of hair, skin, and nails. The three genera of this group, i.e., *Trichophyton, Microsporum*, and *Epidermophyton*, cause significant diseases in human beings and other animals. *Trichophyton* is an anthropophilic species affecting only humans. Among the 40 species of dermatophytes that infect humans, the most common pathogens of scalp infection–tinea capitis are *Trichophyton tonsurans* and *Microsporum canis* (White et al., 2014), and tinea capitis is considered to be the most infectious form of all tinea diseases (Müller et al., 2021).

According to the 2015 World Health Organization (WHO) report, dermatophytes affect ~25% of the world population (Petrucelli et al., 2020), affecting people of all age groups irrespective of whether they are immunocompromised or not (Segal and Elad, 2021). In the past 7–8 years, there has been an acute increase in difficult-to-treat, recurrent and chronic dermatophytosis in developing countries like India. Bongomin et al. (2017) estimated 21,073,423 cases of this infection in just 16 countries, with the majority of these cases in sub-Saharan Africa occurring predominantly in children (Hay, 2017; Nweze and Eke, 2018). In a study conducted in Sikkim, India, ~60.4% of patients gave a history of recurrent dermatophytosis (Sharma et al., 2017).

The infection can be effectively controlled with the use of antifungals, such as azoles and polyenes. However, most of these antifungals are known to cause side effects, such as liver damage, and elicit anaphylactic reactions (Chen et al., 2015). There are also many drug–drug interactions, which are potentially dangerous, especially in immunocompromised patients with conditions such as diabetes, renal failure, and liver dysfunction (Al-Khikani, 2020). In short, most of the clinically employed antifungals suffer from different drawbacks, such as toxicity, drug–drug interactions, no fungicidal effect, and high cost. In addition to this, there is an increase in the occurrence of resistance reported in clinically isolated strains from different regions of the world leading to failure in the treatment of mycoses. A unique multidrug-resistant *Trichophyton* species was isolated in North India showing a high rate of resistance to the three commonly used oral antifungal drugs, i.e., terbinafine, fluconazole, and griseofulvin (Singh et al., 2019). Similarly, terbinafine resistance was detected in the clinical isolates of *T. tonsurans* and *Trichophyton rubrum* at a rate of 2%, indicating the increasing number of antifungal-resistant dermatophytes (Salehi et al., 2018). In addition, there is a serious issue of cross-resistance noticed among the *Trichophyton* strains. For example, terbinafine-resistant *T. rubrum* exhibited cross-resistance to butenafine, tolnaftate, tolciclate, and naftifine (Sinha and Sardana, 2018). Hence, there is a persistent need for newer antifungal agents or formulations that are more effective and less toxic than those which are already in use (Iván et al., 2015; Bajpai et al., 2017).

In recent years, research on the possible use of natural products, such as plant products, beneficial microbes, namely PGPR and probiotics for clinical use, is gaining importance (Sreenivasa et al., 2011; Chennappa et al., 2016; Yooussef et al., 2016; Achar et al., 2020). Probiotics such as lactic acid bacteria (LAB) widely used in the food and pharmaceutical industries are proven to be beneficial and have been used for a number of disease applications and are generally regarded as safe for human use, making them ideal candidates for the development of new therapeutic formulations (Kleerebezem et al., 2017). LAB can potentially produce compounds such as enzymes, vitamins, organic acids, bacteriocins, and other metabolites (Arena et al., 2018). Several of these low molecular weight biomolecules have been isolated and shown to have the potential to inhibit fungal growth (Le Lay et al., 2016; Bukhari et al., 2020). Because LAB are naturally present in many foods (Rawat et al., 2018) and are also associated with health in the mammalian intestine, it is possible that antifungal agents from these do not induce side effects in humans, although this obviously warrants further investigation.

The antifungal activity of probiotic strains, specifically LAB, has been screened with promising results against tinea (Ajah, 2016; Mehdi-Alamdarloo et al., 2016). There are many cases in which probiotics are administered together with conventional drugs clinically for controlling fungal infections such as vulvovaginal candidiasis (Russo et al., 2018), and patents have been obtained for probiotic formulations to be used as topical applications (Angeles and Angeles, 2019). The results obtained from these studies are encouraging and confirm that probiotics and their products are a promising alternative for antifungal treatment.

One of the ideal sources to look for probiotics to fight against indigenous fungal pathogens is the traditional fermented foods prepared locally and have been in use for generations. Different *Lactiplantibacillus* spp isolated from such fermented foods have been examined with success for their antifungal activity (Arena et al., 2018) Although one of the best-known probiotic species, *Lactiplantibacillus plantarum*, has been explored for its antifungal activity against *Aspergillus* spp (Bukhari et al., 2020), *Penicillium* spp (Wang et al., 2012), *Fusarium proliferatum* (Deepthi et al., 2016), and *T. rubrum* (Danial et al., 2021), its effect on *Trichophyton tonsurans* is yet to be investigated.

In the present study, we have analyzed the *in vitro* antifungal activity of a specific probiotic isolate *L. plantarum* MYSN7 from a traditional fermented food, Haria, and have demonstrated the significant effect of the probiotic culture and its cell-free supernatant against *T. tonsurans*.

## Materials and methods

### Preparation of fungal spore suspension

The dermatophyte culture *Trichophyton tonsurans* (MTCC 8475) was procured for this study. The subcultures were grown on Sabouraud dextrose agar (SDA) plates at 30°C for 10 days and preserved at 4°C until further use. *Trichophyton tonsurans* spores were harvested from a 10-day-old culture plate by flooding it with phosphate-buffered saline and gently scraping the surface using a sterile inoculation loop. The spore concentration was adjusted to 10^6^ spores/ml using a hemocytometer for further analysis.

### Preparation of LAB suspension and freeze-dried cell-free supernatant

Haria, a fermented rice beverage that is popular among the rural tribe of West Bengal and East-Central India (Bengal et al., 2014), was prepared in the laboratory by inoculating cooked and dried rice with the starter culture bakhar, which is collected from five different sources. The inoculated rice sample was fermented for 14 days at room temperature. The fermented liquor was further used for the isolation of LAB adopting the method of Rao et al. (2015). The sample was first enriched in de Man Rogosa Sharpe (MRS) broth under anaerobic conditions at 37°C for 24 h. The enriched stock was then serially diluted using phosphate-buffered saline, spread on MRS agar plates, and incubated under the same conditions. The discrete colonies were further sub-cultured onto MRS agar plates.

Each LAB strain was maintained in glycerol stock. To obtain the cell-free supernatant (CFS), 300 μl of LAB cell suspension (10^6^ CFU/ml) was inoculated in 30 ml of MRS broth and incubated at 37°C for 48 h. The CFS was obtained by centrifuging the broth at 3,000 rpm for 15 min at 4°C. It was then sterilized by filtration using a 0.2-μm syringe filter (AXIVA) and freeze-dried aseptically (Danial et al., 2021).

### LAB preliminary identification and characterization

Preliminary identification was performed through microscopic analysis and biochemical tests including gram staining, catalase test, bile salt hydrolysis (0.3% ox gall), osmotic stress tolerance (3, 5, and 7% NaCl), and carbohydrate fermentation. The growth of the isolates at different temperatures (4, 10, 37, and 45°C) was conducted and recorded (Ni et al., 2015).

### Screening of LAB for its antifungal activity

The antifungal activity of the LAB isolates was examined using the agar overlay method against *T. tonsurans*, as per the procedure of Quattrini et al. (2018). Log phase MYSN7 culture was streaked as 2-cm lines on MRS agar plates and incubated at 37°C for 24 h. Then, 1 ml of conidial suspension (10^6^ spores/ml) was mixed with 100 ml molten and cooled SDA medium containing 0.7% agar. The medium containing spore suspension was gently overlaid on the MRS plates with bacterial streaks, maintained undisturbed for solidification, and then incubated at 30°C for 7 days. The activity was graded based on the diameter of the zone of inhibition (+++ >8%−10% area of the plate showing zone of inhibition, ++ 3–8% of the plate showing zone of inhibition, + visible inhibition only above the streak, and – no visible inhibition). The bacterial isolate showing the maximum zone of inhibition was taken for further studies.

### Molecular characterization

The LAB isolate MYSN7 with potential antifungal activity was identified by 16S rRNA amplification followed by Sanger sequencing. DNA isolation was carried out using the phenol–chloroform–isoamyl alcohol method. The purified DNA was amplified by PCR using the primer pairs 8F-(5′GAGTTTGATCCTGGCTCAG3′) and 1391R-(5′ GACGGGCGGTGWGTRCA3′) (Lane et al., 1985). Following identification, a phylogenetic tree was constructed using the MEGA-X software by neighbor-joining method. The sequence has been submitted to NCBI GenBank and an accession number was obtained.

### Determination of growth kinetics

The isolate MYSN7 was inoculated in 15 ml MRS broth and incubated overnight. Furthermore, it was transferred to a fresh medium in a 96-well plate, and the absorbance was measured every 2 h for 24 h at 600 nm. The readings were taken and graphically represented (Somashekaraiah et al., 2021).

### Determination of probiotic activity assays

#### Acid tolerance and bile tolerance

The isolate's ability to tolerate the gastric pH and bile was tested as per the protocol of Yadav et al. (2016) with minor modifications. For this, overnight culture of the LAB was inoculated in two sets of MRS broth tubes, which have been adjusted to pH 2 and pH 3. Similarly, broth containing 0.3% ox gall was inoculated with the same culture. All the tubes were incubated at 37°C. Samples were withdrawn each hour for 3 h, and OD_600_ was measured. The survival percentage of MYSN7 was calculated using the formula (OD test/OD control) × 100.

#### Auto-aggregation

The isolate grown overnight was centrifuged at 8,000 rpm for 10 min, and the pellet was resuspended in 5 ml of PBS adjusting the OD to 0.25 ± 0.05 at 600 nm. The sample was allowed to stand at 37°C, and the upper suspension was checked for absorbance (OD_600_) at intervals of 0, 1, 2, 3, and 4 h. The aggregation was measured by applying the formula auto-aggregation % = [1 – (*A*_t_/*A*_0_) × 100], where A_t_ represents the absorbance at a particular time and A_0_ represents the initial absorbance (Somashekaraiah et al., 2019).

#### Cell surface hydrophobicity

MYSN7 overnight culture was suspended in 3 ml of PBS and adjusted to OD_600_ of 0.25 ± 0.05. Then, 1 ml of xylene was added, mixed gently, and kept undisturbed at 37°C for phase separation. After every hour for 3 h, the lower aqueous phase was drawn and OD_600_ was measured. The percent of hydrophobicity was calculated using the formula [(*A*_0_ – *A*_t_/*A*_0_)] × 100, where *A*_0_ is the initial absorbance and *A*_t_ is the absorbance of the aqueous phase at time *t* (Rokana et al., 2018).

#### Antibiotic susceptibility profile

Probiotic isolates should meet the safety standards for application in food and humans. One such assay that is routinely carried out is the antibiotic susceptibility profile to the commonly used antibiotic as given in EFSA guidelines. The sensitivity pattern of the MYSN7 to these antibiotics was tested by the Kirby–Bauer disc diffusion procedure. The diameter of the zone of inhibition (ZOI) was measured, and the antibiotic sensitivity pattern was interpreted as sensitive, intermediate, or resistant following the CLSI (2014) guidelines.

#### Antibacterial activity

This was assessed by the microtiter plate technique using the method described by Somashekaraiah et al. (2019). The CFS of the isolate MYSN7 was tested against the common pathogens *Escherichia coli* (ATCC 25922), *Pseudomonas aeruginosa* (ATCC 15422), *Staphylococcus aureus* (ATCC 6538), *Klebsiella pneumoniae* (MTCC 7407), and *Salmonella paratyphi* (ATCC 9150). Then, 50 μl of the sterile CFS and 50 μl of the bacterial suspension were added to the wells with the final cell concentration of ~10^8^ CFU/ml. It was made up to 200 μl using the Luria–Bertani broth and incubated at 37°C. Wells having uninoculated LB broth and broth inoculated with bacterial suspension were maintained as negative and positive controls, respectively. OD was recorded at 600 nm after 24 h of incubation, and the percentage inhibition of growth was calculated using the formula [(OD_Control_ – OD_Test_)/OD_Control_] × 100.

#### Hemolytic activity

This test was performed for observing the hemolytic activity of the isolate. The overnight culture was streaked on the blood agar plate (5% sheep blood) and incubated at 37°C for 24 h. *Staphylococcus aureus* (MTCC 87) was streaked on another blood agar plate as a positive control. The plates were observed for α (partial), β (complete), or γ (no) hemolysis (Yadav et al., 2016).

### Analysis of antifungal activity

#### Co-inoculation assay

To determine the effect of MYSN7 on the growth kinetics of *T. tonsurans*, the co-inoculation assay as described by Deepthi et al. (2016) was performed. Modified MRS medium (mMRS) was prepared according to the composition given (bacteriological peptone 5 g/L, mycological peptone 5 g/L, beef extract 10 g/L, yeast extract 5 g/L, dextrose 20 g/L, MgSO_4_ 0.10 g/L, MnSO_4_ 0.05 g/L, and K_2_HPO_4_ 2 g/L). The medium is devoid of antifungal substances, such as sodium acetate, polysorbate 80, and ammonium citrate. Then, 100 μl of overnight LAB culture (10^6^ cfu/ml) and 100 μl of conidial suspension (10^6^ spores/ml) were inoculated in 50 ml of mMRS broth. At different intervals of incubation (3, 7, 10, and 14 days), the mycelial mass was separated by filter paper and the weight was measured after drying at 80°C. Then, 50 ml of mMRS broth with only the mold spores was kept as a control. The experiment was performed in triplicates.

### Fungal biomass inhibition by CFS-MYSN7

A biomass inhibition assay was determined using the protocol described by Quattrini et al. (2018). Then, 50 ml of sterile SDB was supplemented with different concentrations of sterile CFS (2%, 4%, 6%, 8%, and 10%), inoculated with *T. tonsurans* spores (10^6^ spores/ml), and incubated at 30°C. Then, 50 ml of SDB inoculated with spores was maintained as a control. After 10 days, the percentage of inhibition was calculated by comparing the weight of the mycelial mat with that of the control.

### Conidial germination inhibition assay

The effect of CFS-MYSN7 on the conidial germination of *T. tonsurans* was determined using a 24-well microtiter plate adopting the method of Guo et al. (2011) with some modifications. In the well, 200 μl of CFS-MYSN7 and 100 μl of conidial suspension (10^6^ spores/ml) were added and made up to 1 ml using SDB. The well containing the fungal spores and the broth medium without CFS was kept as a control. The plate was incubated at 30°C, and the conidial germination was observed at 2, 4, 8, 16, 24, 48, and 72 h by mounting 10 μl of sample on a microscopic slide and observed under the phase contrast microscope (Carl Zeiss AXIO, Germany) with bright-field illumination. Germinated conidia were counted using a hemocytometer, and the percentage of conidial germination was calculated by applying the formula: [number of germinated conidia/total conidia counted] × 100.

### Determination of MIC

Freeze-dried CFS powder (CFSp) of LpMYSN7 was prepared to find the minimum inhibitory concentration against *T. tonsurans*. The standard antifungal drug ketoconazole (HI Media, Mumbai, CMS4322) was used as a positive control. Minimum inhibitory concentration (MIC) determination of the CFSp-LpMYSN7 was carried out following the procedure of EUCAST antifungal MIC methods for molds (EUCAST E.DEF 9.3 December 2015) using the broth microdilution method. For MIC determination, 10 μl of conidial suspension (10^4^ spores/ml) was taken in a 96-well microtiter plate, and 190 μl of different concentrations of CFSp (8 mg/ml−0.0625 mg/ml) prepared using the serial double dilution method was added. Similarly, the procedure was repeated for the standard drug ketoconazole with concentrations of 8–0.065 μg/ml. The plate was incubated at 30°C for 72 h before observing the growth inhibition. MIC was determined as the lowest concentration of the extract inhibiting visual growth of the test cultures. Three replications were conducted to confirm antifungal activity (Arasu et al., 2014).

### Residual bioactivity of CFS-MYSN7

To understand the chemical nature of the antifungal compound in the CFS, the effect of different factors such as heat, pH, proteases, extended storage, and freeze–thaw cycle on the CFS-LpMYSN7 was performed following the method of Kang et al. (2011) with modifications. To determine the effect of heat, the CFS was kept in an 80°C water bath for 20 min and cooled. To nullify the effect of bacteriocin-like compounds, 5 ml of CFS was treated with proteinase-K (1 mg/ml) and incubated for 3 h at 37°C and the enzyme was then heat-inactivated at 80°C for 5 min (Jamwal et al., 2019). The pH of the CFS was adjusted to 7 using sterile 1 M NaOH to neutralize the antifungal activity of organic acids present. To test the effect of freeze–thaw cycle, 1 ml of CFS was frozen at −20°C for 24 h and thawed for 20 min at 4°C (Ponce et al., 2008). To know the efficiency of the antifungal activity of CFS after long-term storage, it was kept stored at −20°C for 2 months before use.

All the above-treated CFS were tested for residual anti-*Trichophyton* activity using the microdilution method using 24-well polystyrene flat-bottomed cell culture plate (NEST Biotechnology Co., Ltd, China). Then, 200 μl of treated CFS was mixed with 100 μl conidial suspension (10^6^ spores/ml) and made up to 1 ml using SDB medium. The well containing conidial suspension with SDM alone was kept as control. The plate was incubated at 30°C and observed visually for growth inhibition after 7 days.

### Organic acid profiling of the CFS*-*MYSN7 using LC-MS/MS

The extraction of organic acids was performed as described by Ribeiro et al. (2007). An aliquot of 500 μl of the sample was made up to 5 ml using 80% methanol. From this, 0.1 ml was taken and made up to 1 ml with mobile phase and then filtered, and 4 μl of this was injected into the LC-MS/MS (Waters UPLC H class system fitted with TQD-MS/MS system) for further analysis. The analytical column used was a 2.1 × 50 mm UPLC BEH-Amide column (Waters) with 1.7 μm particles, protected by a Vanguard 2.1 × 5 mm BEH-Amide with 1.7 μm particle size guard column (Waters). The column temperature was maintained at 25°C. The sample injection volume was 4 μl. The eluted organic acids were monitored using the TQD-MS/MS (Waters, USA) system, which was optimized for the detection and quantification of organic acids analysis (Kuwaki et al., 2002).

### Scanning electron microscopic analysis

To observe the effect of MYSN7 on the mycelial morphology of *T. tonsurans*, SEM analysis was performed on treated and untreated fungal mycelial structures (Chandra, 2013). Fungal mycelia were picked from both treated and control plates for the study. The dehydrated mycelia were mounted on an aluminum stub with carbon tape, coated with gold–palladium alloy, and observed under the EVO LS 15 Scanning Electron Microscope (Carl Zeiss, Germany).

### Statistical analysis

The data obtained from this study were the mean of three replicates expressed as mean ± standard deviation and analyzed using two-way analysis of variance (ANOVA) using the software GraphPad Prism version 8.0.2 (263) (GraphPad Software Inc.). Values of *p* < 0.05 were considered to be statistically significant.

## Results

### Preliminary identification and characterization of LAB

A total of 20 LAB strains were isolated from Haria—the fermented rice beverage. The chosen LAB isolates were Gram-positive, rod-shaped, and catalase negative. The three strains that survived the preliminary screening for LAB included MYSN6, MYSN7, and MYSY1. The physiological characteristics of these three strains have been tabulated (Table 1).

**Table 1:** Preliminary characterization of LAB strains.

**Assays**	**Isolates**		
	**MYSN6**	**MYSN7**	**MYSY1**
Gram's staining	+	+	+
Morphology	Rod	Rod	Rod
Catalase test	–	–	–
Citrate utilization test	–	–	–
Bile salt hydrolysis (0.3%)	+	+	+
**Growth at NaCl**			
3%	+	+	+
5%	+	+	+
7%	+	+	+
**Growth of isolates at various temperature**			
4°C	–	–	–
10°C	+	+	+
37°C	++	++	++
45°C	+	+	+
Fermentation of sugars	Hetero	Hetero	Hetero
D-Glucose	++	++	+
D-Arabinose	+++	+++	+++
Lactose monohydrate	+++	+++	+++
D-Sorbitol	+++	+++	+++
D-Raffinose	+++	+++	+++
Mannitol	+++	+++	++
D-Xylose	++	++	+
L-Arabinose	++	++	++
D-Maltose	+++	+++	+++
D-Fructose	+++	+++	+++

+ (weak growth); ++ (good growth); +++ (very good growth); – (no growth); hetero, heterofermentative.

### Screening of antifungal activity

**Figure 1:**
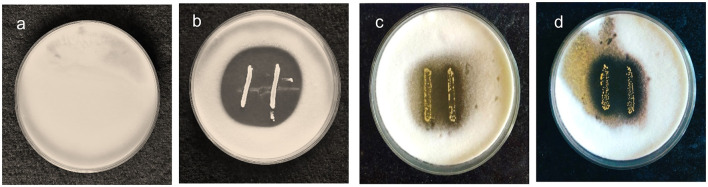
Agar overlay plates showing the growth inhibition of *T. tonsurans* in the presence of probiotic isolates. **(a)** Control plate of *T. tonsurans*. **(b)** Antifungal activity of MYSN7. **(c)** Antifungal activity of MYSN6. **(d)** Antifungal activity of MYSY1.

The antifungal activity of the chosen strains was examined using the agar overlay method and was graded based on the diameter of the inhibition zone. The activity of MYSN7 against *T. tonsurans* was graded as +++ as the zone of inhibition covering the area of >8%−10% of the agar plate, whereas the activity of MYSN6 and MYSY1 was graded as ++ as the zone of inhibition covered was 3%−8% of the agar plate. Based on the observation, the LAB isolate MYSN7 was selected for further analysis (Figure 1).

### Molecular characterization

**Figure 2:**
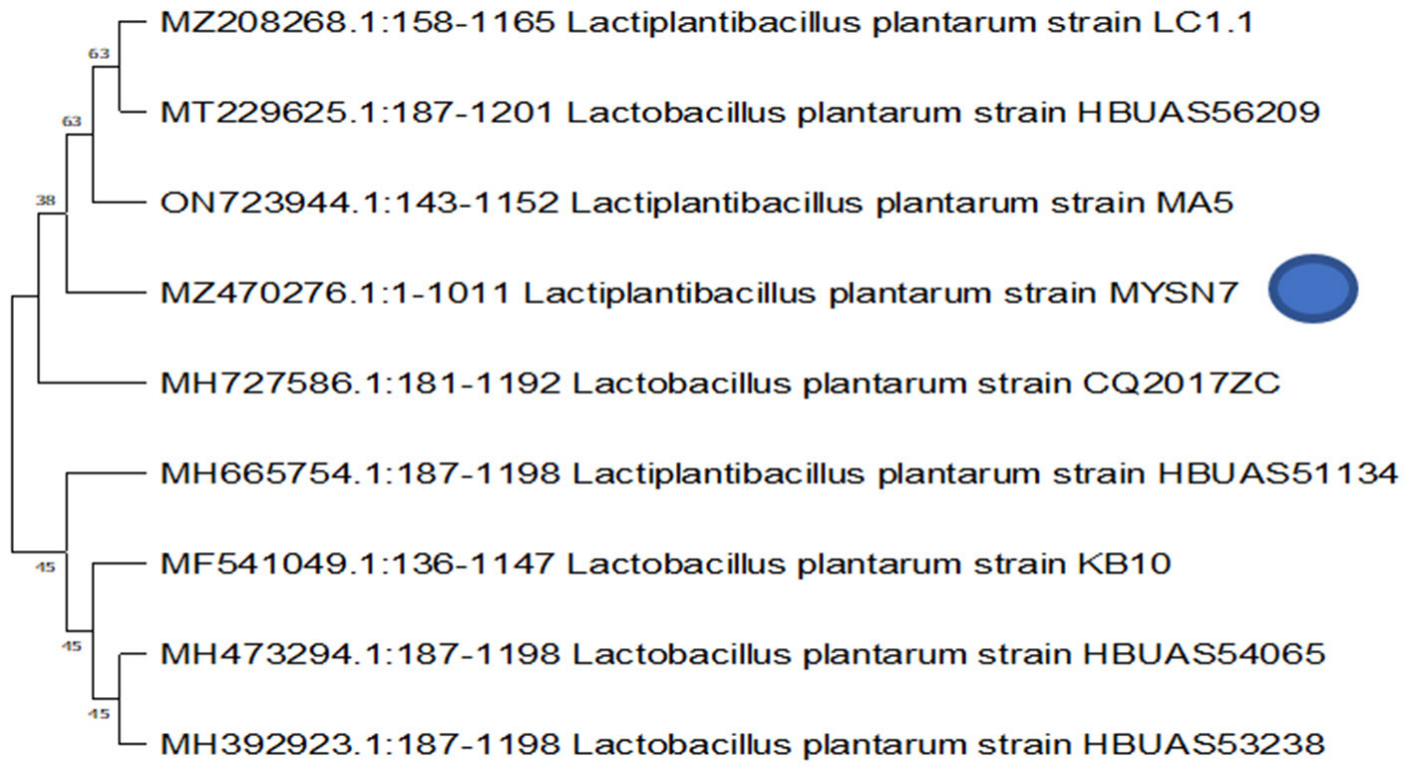
Molecular characterization and phylogenetic analysis of MYSN7. Phylogenetic tree showing the relationship between different *Lactobacillus* spp. The dendrogram was constructed based on the neighbor-joining method.

MYSN7 isolate showing maximum antifungal activity was identified using a molecular method. The isolated genomic DNA was quantified using nanodrop and found to be 733 ng/μl. The quality of the PCR amplified product was checked with agarose gel electrophoresis. By 16S rRNA sequencing, the isolate MYSN7 was found to be *L. plantarum*, and the sequence was deposited in NCBI GenBank with the accession number MZ470276. The phylogenetic tree constructed using the neighbor-joining method is shown in Figure 2.

### Growth kinetics of *Lp*MYSN7

**Figure 3:**
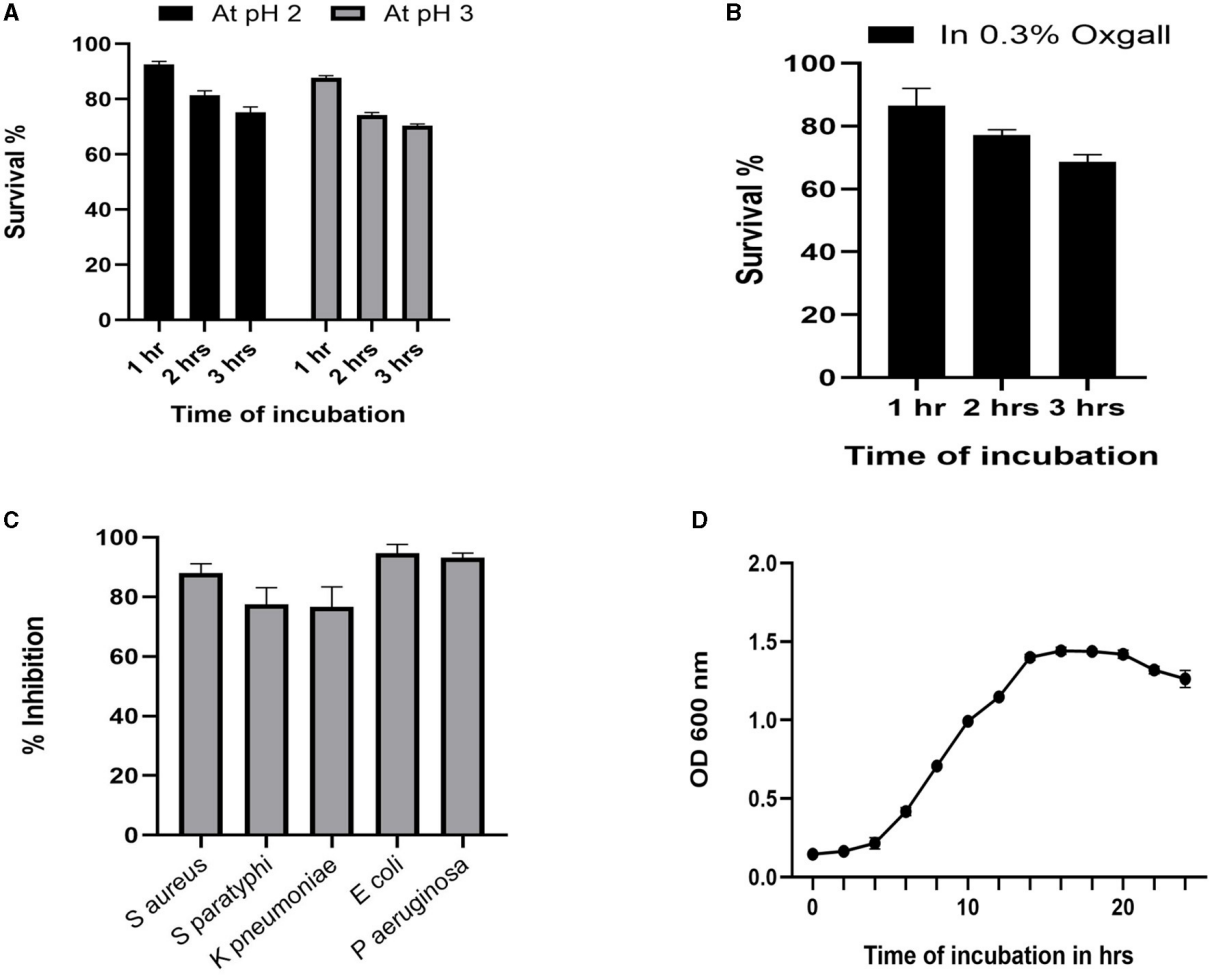
Probiotic properties of *Lp*MYSN7. **(A)** Survival percentage of the isolate at pH2 and pH3. **(B)** Survival percentage of the isolate in the presence of 0.3% ox gall. **(C)** Antibacterial activity of LpMYSN7 CFS against the common pathogens. **(D)** Growth curve of LpMYSN7 with logarithmic growth phase from 4 to 14 h. Data shown are mean ± SE of triplicate values of independent experiments and differ significantly (*p* < 0.0001).

The growth pattern of the identified strain *L. plantarum* MYSN7 (LpMYSN7) was analyzed by plotting the graph with the *Y*-axis showing the OD of the growing culture at 600 nm and the *X*-axis showing the time of incubation in hours (Figure 3D). For the first 2–3 h, there was no increase in the OD value, and after 4 h of incubation, there was a logarithmic increase in the growth of this bacterium that continued for 14 h. After this, there was a stationary period of 6 h and a subsequent decrease in the OD for the last 2 h of incubation.

### Probiotic attributes of LpMYSN7

Studying the probiotic attributes of the LAB isolates helps in the *in vitro* assessment of the survival and colonization of the isolate in the digestive system. LpMYSN7 showed an average survival percentage of 75% in pH 3 and a 70% survival percentage in pH 2 after 3 h of incubation when compared to the control (Figure 3A). The percentage of tolerance to 0.3% ox gall after 3 h of incubation was 68.73% (Figure 3B). The isolate showed moderate hydrophobicity of 48.87% after 3 h of incubation with the hydrocarbon xylene, and the auto-aggregation percentage was 80.62% after 4 h of incubation.

### Antibiotic susceptibility of LpMYSN7

**Figure 4:**
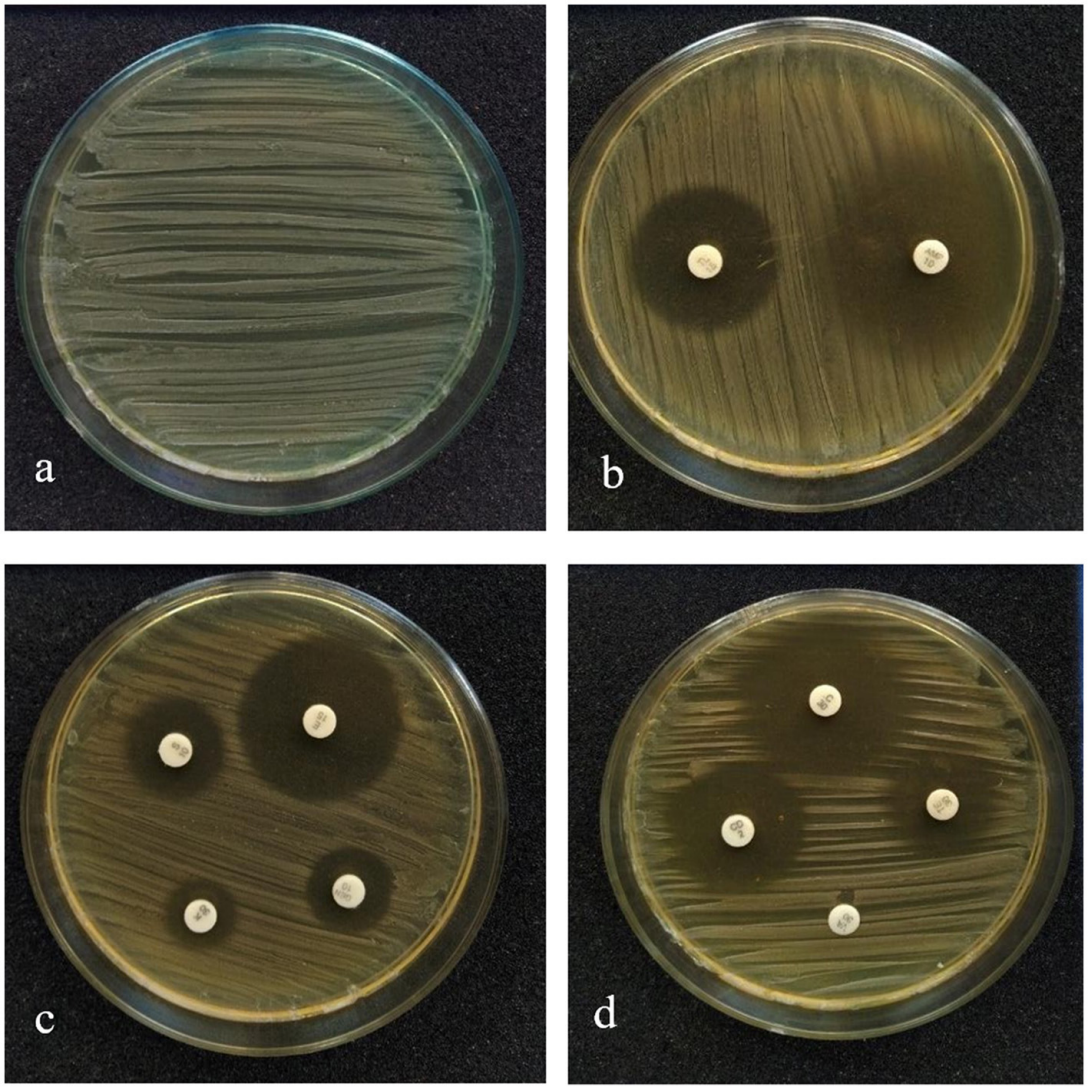
Antibiotic sensitivity pattern of LpMYSN7. **(a)** Control plate. **(b)** ZOI for ampicillin and gentamycin. **(c)** ZOI for kanamycin, tylosine, and clindamycin. **(d)** ZOI for tetracycline, erythromycin, chloramphenicol, and streptomycin.

The susceptibility profile of the LAB isolate to the commonly used antibiotics needs to be assessed before it is employed for human use. The antibiotics that were used for this study and the sensitivity pattern of LpMYSN7 are shown in Table 2. The sensitivity characteristic of the organism was determined by measuring the diameter of the ZOI and referring to the interpretative chart given in the catalog. It was found to have sensitivity toward ampicillin, gentamycin, kanamycin, chloramphenicol, erythromycin, clindamycin, tetracycline, and tylosine. There is moderate sensitivity for streptomycin, and the organism is resistant to vancomycin (Figure 4).

**Table 2:** Antibiotic sensitivity profile of *L. plantarum* MYSN7.

**Antibiotic used**	**Zone of inhibition (mm)**	**Sensitivity pattern**
Ampicillin (10 μg/disc)	38.5	Sensitive
Vancomycin (30 μg/disc)	0	Resistant
Gentamycin (10 μg/disc)	17	Sensitive
Kanamycin (30 μg/disc)	13	Sensitive
Streptomycin (10 μg/disc)	17.5	Moderately sensitive
Chloramphenicol (30 μg/disc)	30.5	Sensitive
Erythromycin (15 μg/disc)	30.5	Sensitive
Clindamycin (2 μg/disc)	24.5	Sensitive
Tetracycline (30 μg/disc)	21.5	Sensitive
Tylosine (15 μg/disc)	27.5	Sensitive

### Antibacterial activity

The cell-free supernatant of LpMYSN7 shows strong antibacterial activity against common enteric pathogens as shown in Figure 3C. The inhibition percentages were 88.01%, 77.45%, 76.59%, 94.68%, and 93.17% against *S. aureus, S. paratyphi, K. pneumoniae, E. coli, and P. aeruginosa*, respectively.

### Safety evaluation

**Figure 5:**
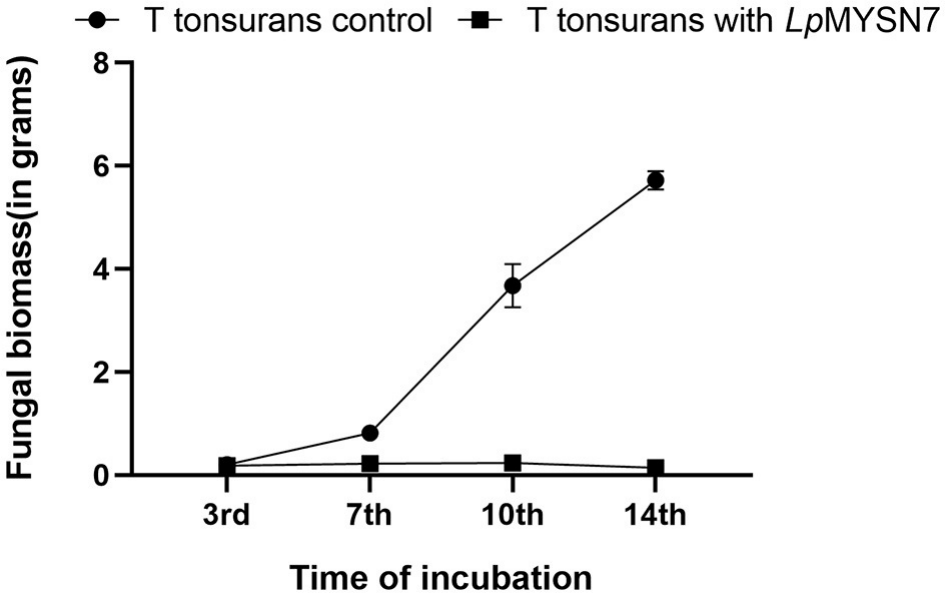
Growth rate of *T. tonsurans* with and without LpMYSN7 at different time intervals: 3, 7, 10, and 14 days of incubation. Data shown are mean ± SE of triplicate values of independent experiments and differ significantly (*p* < 0.0001).

Hemolytic activity of the probiotic isolate was observed on the blood agar plate to confirm its safety. It showed no hemolysis, which is an indication of its avirulent character when compared to the control strain *S. aureus* (MTCC 87), which showed β hemolysis on the blood agar plate (Figure 5).

### Anti-*Trichophyton* activity of LpMYSN7

#### *Trichophyton tonsurans* inhibition by LpMYSN7 in co-inoculation

**Figure 6:**
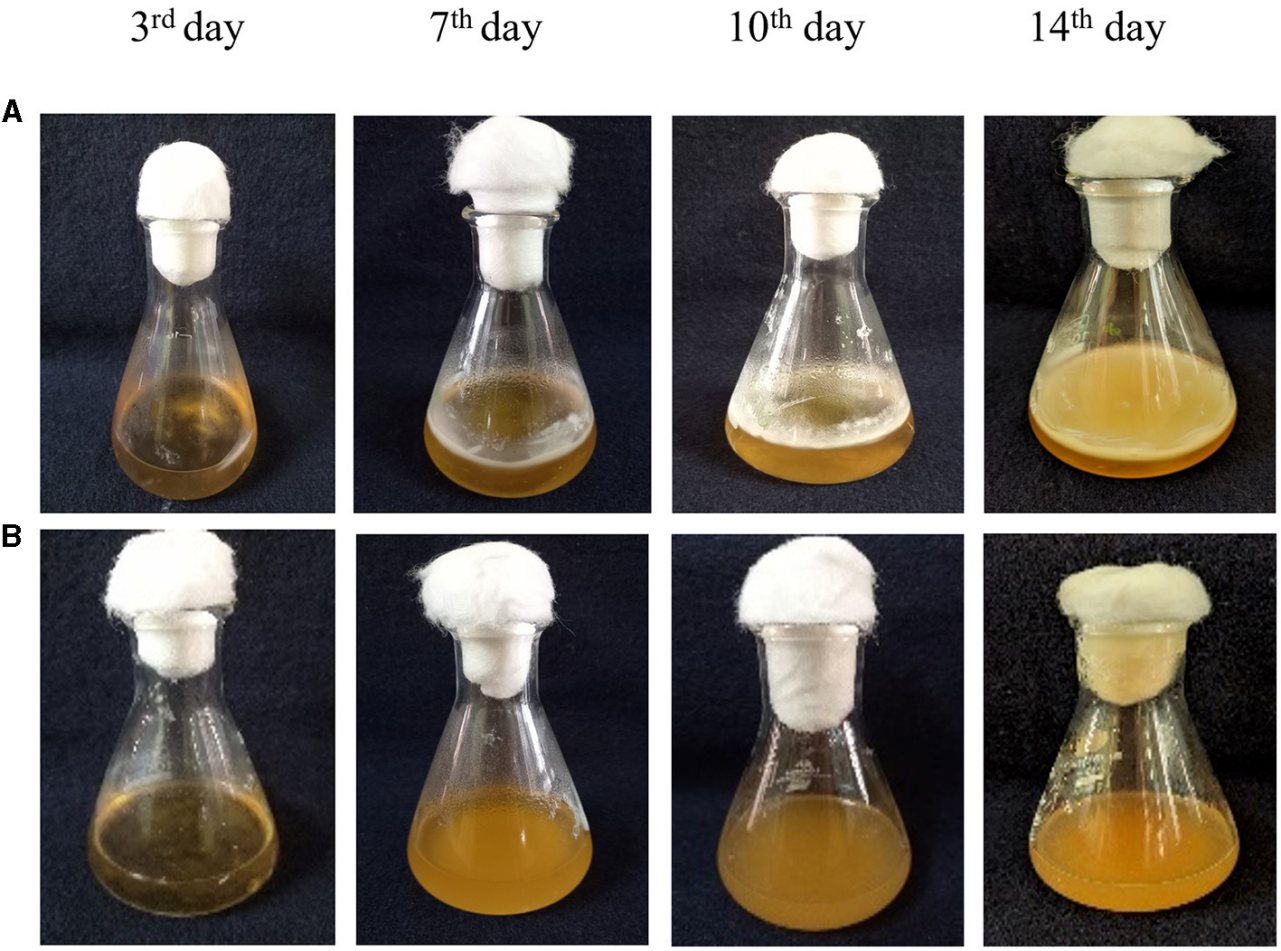
Co-inoculation of LpMYSN7 with *Trichophyton tonsurans* spores. **(A)** Growth of *T. tonsurans* (control) showing a dense mycelial mat after 14 days of incubation. **(B)** *T. tonsurans* showing no significant increase in growth when inoculated with LpMYSN7.

Growth inhibition studies in liquid culture were carried out by co-inoculation of the *T. tonsurans* spores and the cells of LpMYSN7 in modified MRS broth. Biomass of fungal culture was measured after 3, 7, 10, and 14 days. It was found that *T. tonsurans* culture biomass increased constantly over the 14-day incubation period from 0.201 to 5.720 g in the control flask but showed no significant growth (*p* < 0.001) when grown with *Lactiplantibacillus* culture (Figures 5, 6), whereas the bacteria were found to survive throughout the incubation period with an initial increase in the concentration of cells followed by a steady decline of cells with increasing incubation time (Table 3).

**Table 3:** Growth kinetics of *T. tonsurans* and LpMYSN7 co-inoculated in modified MRS broth.

**Days**	**Biomass (g)**		**Log CFU ml** ^**−1**^	
	**Tt control**	**Tt** + **LpMYSN7**	**LpMYSN7 control**	**Tt** + **LpMYSN7**
3	0.20 ± 0.01	0.18 ± 0.01	9.22 ± 0.05	9.16 ± 0.04
7	0.82 ± 0.01	0.22 ± 0.02	9.23 ± 0.07	9.09 ± 0.18
10	3.67 ± 0.42	0.24 ± 0.00	6.09 ± 0.10	5.91 ± 0.18
14	5.72 ± 0.17	0.14 ± 0.03	4.09 ± 0.05	3.97 ± 0.11

n = 3.

Mean ± SD; Tt, Trichophyton tonsurans; LpMYSN7, Lactiplantibacillus plantarum MYSN7.

#### Biomass inhibition of *T. tonsurans* by CFS-LpMYSN7

**Figure 7:**
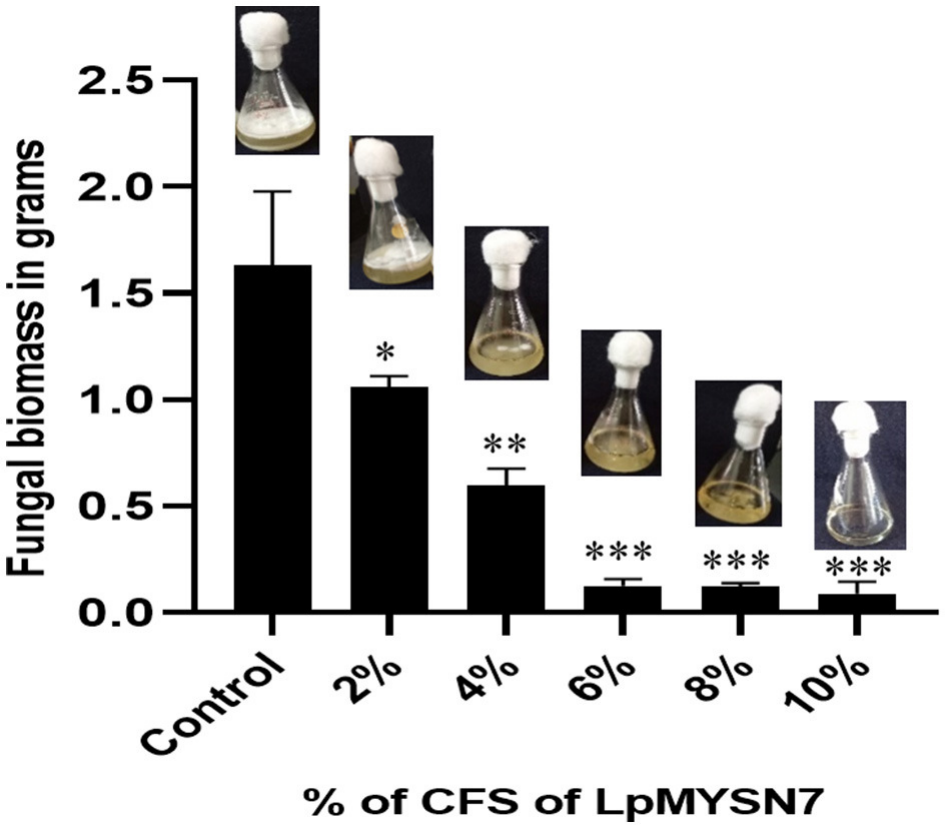
Biomass inhibition of *Trichophyton tonsurans* after 10 days of incubation in the presence of different concentrations (2%, 4%, 6%, 8%, and 10%) of sterile CFS-LpMYSN7 compared with the positive control. Data shown are mean ± SE of triplicate values of independent experiments and differ significantly (*p < 0.05; **p < 0.01; ***p < 0.001).

When the biomass of dermatophyte culture grown in SDB with different concentrations of CFS of the LpMYSN7 was weighed after 10 days of incubation, a gradual reduction in mycelial growth of 1.058, 0.594, 0.125, 0.123, and 0.089 g was observed with 2%, 4%, 6%, 8%, and 10% CFS, respectively, whereas the biomass of the control was 1.632 g after the same period of incubation (Figure 7).

### Conidial germination inhibition

**Figure 8:**
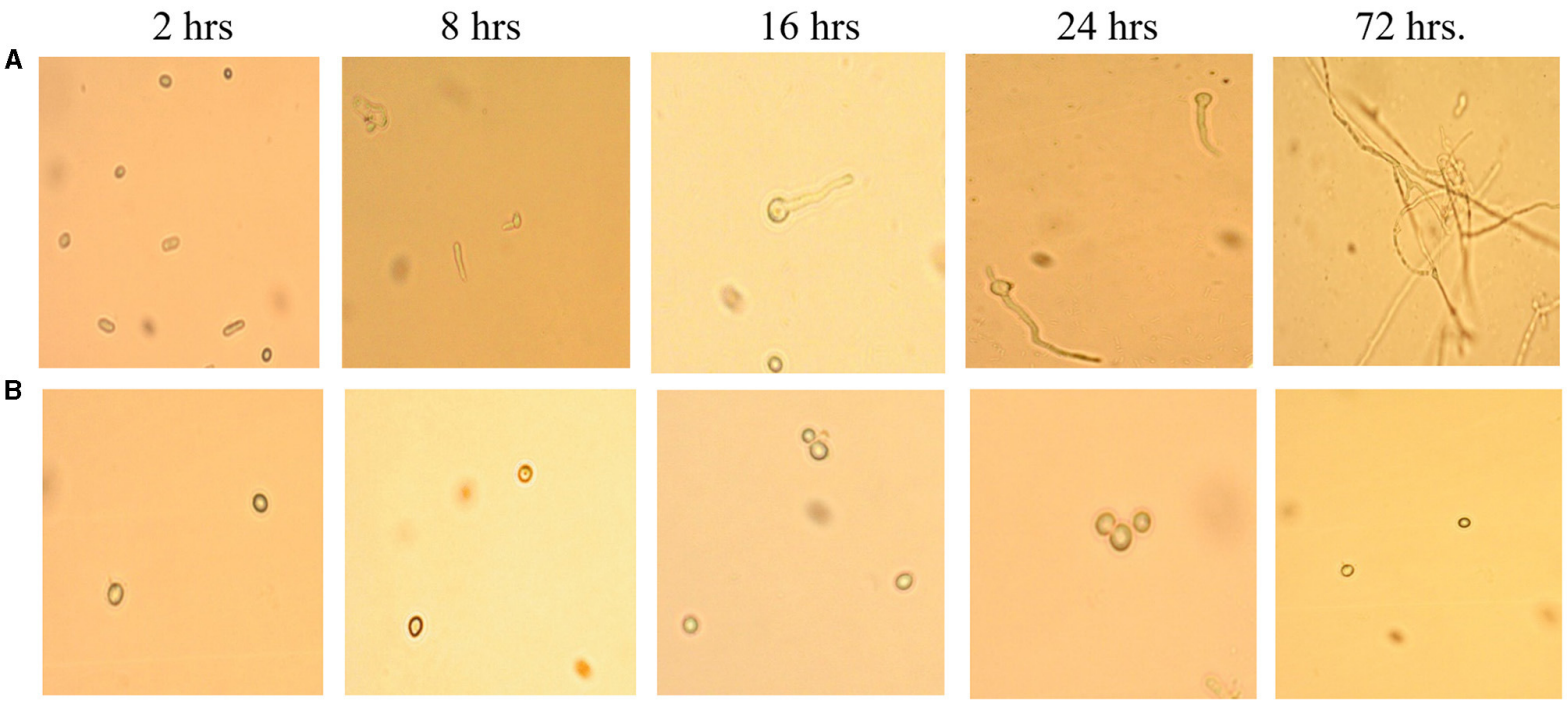
Effect of CFS-LpMYS7 on the conidial germination of the fungal pathogen. **(A)** Control—Conidia of *T. tonsurans* showing germination at 2, 8, 16, 24, and 72 h. **(B)** Conidia treated with CFS-LpMYSN7 showing retarded germination at the same time intervals.

**Figure 9:**
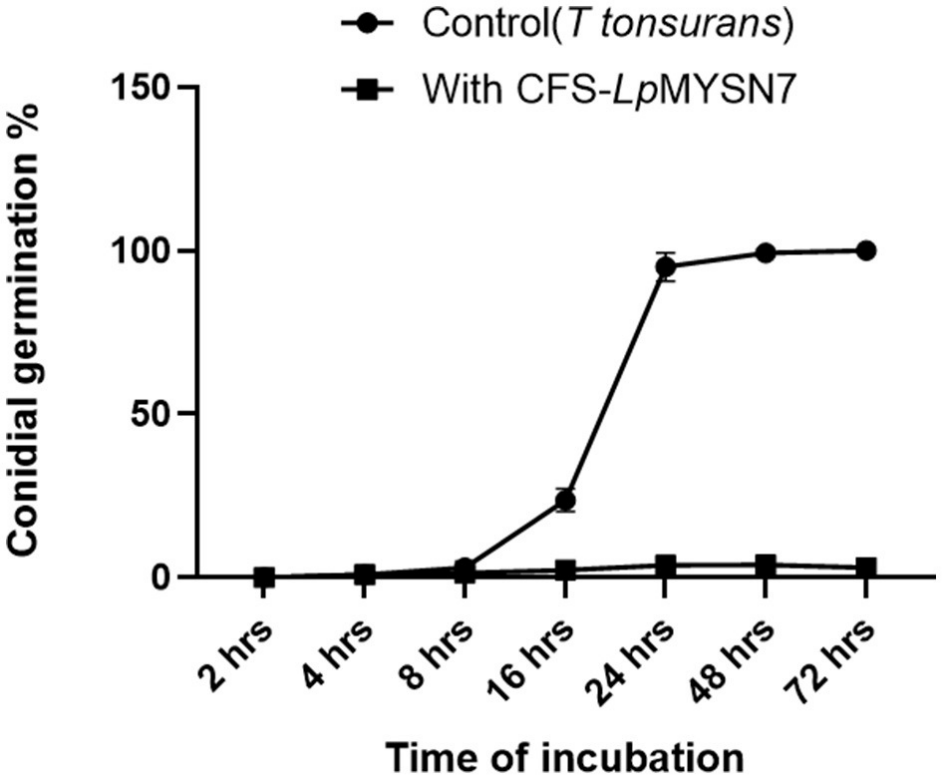
Percentage of conidial germination of *T. tonsurans* control and the conidia treated with CFS-LpMYS7 at different time intervals of 2, 4, 8, 16, 24, 48, and 72 h. Data shown are mean ± SE of triplicate values of independent experiments and differ significantly (*p* < 0.0001).

This assay showed inhibition of conidial germination with CFS-LpMYSN7 when observed at different intervals of time for 3 days. In the control, the germination of conidia was initiated at 16 h and reached a maximum of more than 95% after 48 h of incubation, and at 72 h, 100% germination was observed (Figure 8A), whereas, in CFS-treated conidia, the germination was negligible even after 72 h of incubation exhibiting the effective control of conidial germination (*p* < 0.001) by CFS-LpMYSN7 (Figure 8B). The percentage of conidial germination of *T. tonsurans* conidiospores inoculated in CFS-treated SDB was negligible with 0, 8.69 ± 0.9, 3.92 ± 0.3, 6.67 ± 0.3, 4.55 ± 0.7, 4.76 ± 0.3, and 2.22 ± 1.4 germination percentage at 2, 4, 8, 16, 24, 48, and 72 h, respectively (Figure 9).

### MIC determination

**Figure 10:**
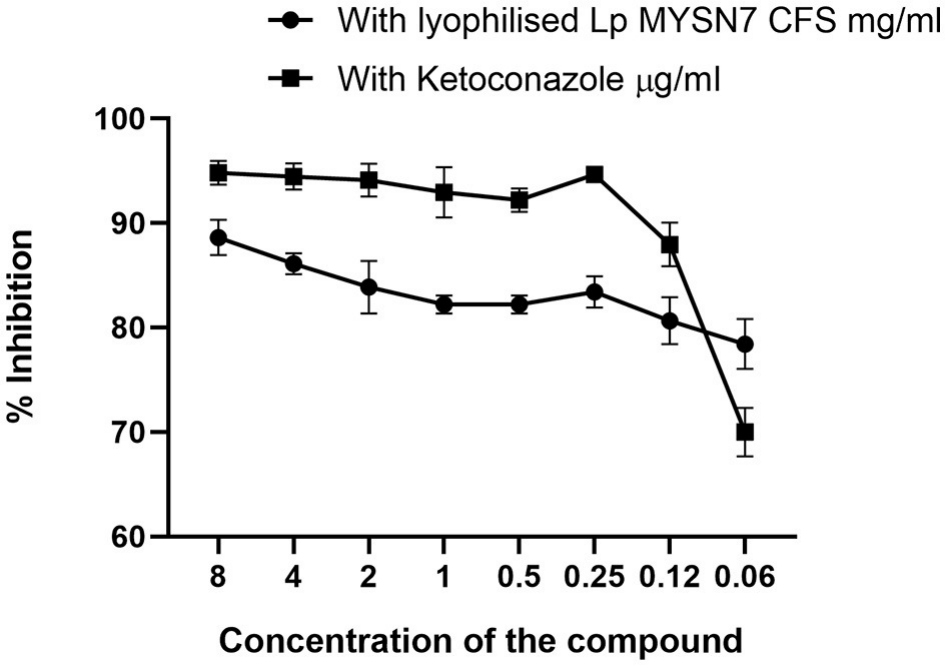
Inhibition of *T. tonsurans* in different concentrations of lyophilized CFS-LpMYSN7 and ketoconazole. Data shown are mean ± SE of triplicate values of individual experiments and differ significantly (*p* < 0.0001).

The anti-*Trichophyton* activity of lyophilized extract of CFS-LpMYSN7 (CFSp) was carried out with different concentrations of the dried extract (8–0.0625 mg/ml). CFSp showed promising activity, with a MIC value of 8 mg/ml against *T. tonsurans*. At this concentration, visual growth of the fungi could not be observed in the microtiter plate, and the conidial germination inhibition was found to be ~88.6 ± 1.6%. The inhibition percentage kept decreasing at increasing dilutions of the lyophilized extract, which has been plotted in the graph (Figure 10). The anti-*Trichophyton* activity of CFSp was compared with the standard antifungal drug ketoconazole with concentrations of 8–0.06 μg/ml, and the MIC value for the same was 1.0 μg/ml, showing conidial germination inhibition of 92.9 ± 2.3%.

### Characterization of CFS-LpMYSN7

**Figure 11:**
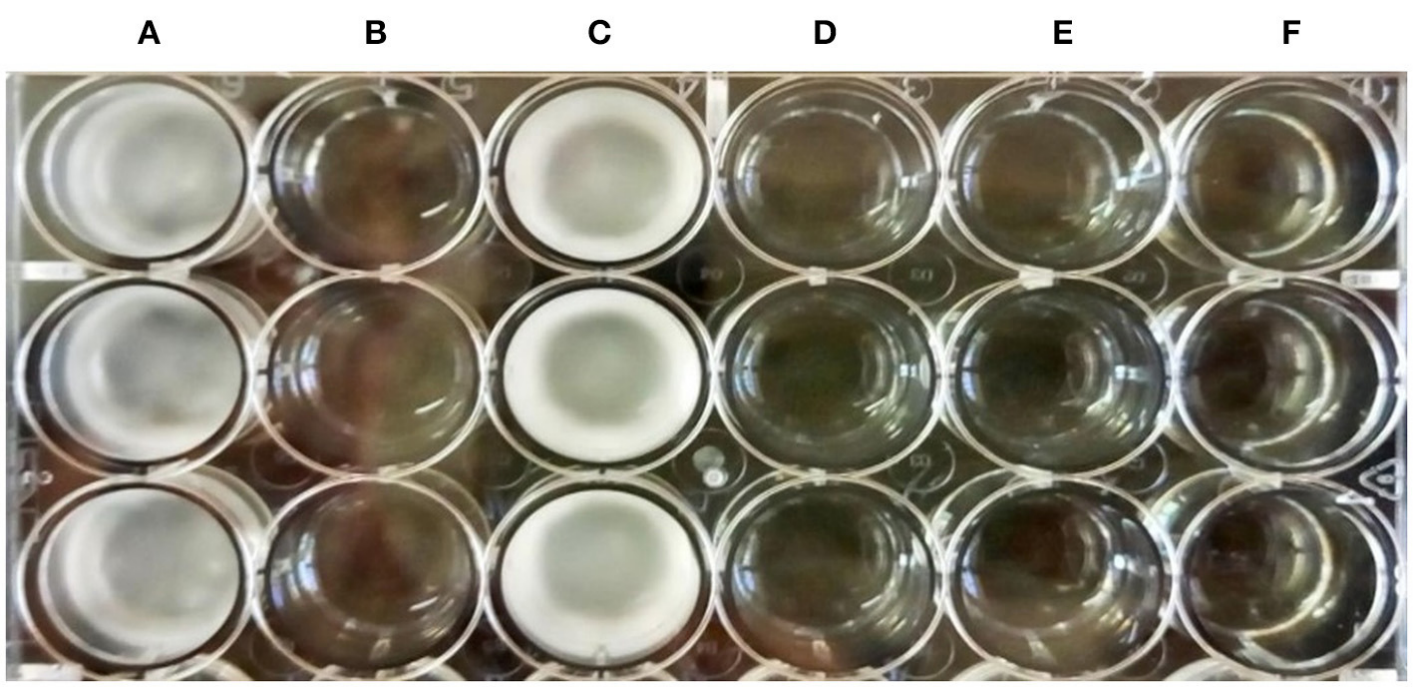
Antifungal activity of the treated CFS-LpMYSN7 against *T. tonsurans*. **(A)** Control well with SDB inoculated with the pathogen. **(B)** Heat-treated CFS + Pathogen showing no growth. **(C)** Neutralized CFS + Pathogen. **(D)** Proteinase-K-treated CFS + Pathogen. **(E)** Frozen and thawed CFS + Pathogen. **(F)** Long-term-stored CFS + Pathogen.

When the CFS was subjected to heat treatment, proteinase-K treatment, freeze–thaw cycle, or long-term storage, its antidermatophytic activity was unaffected as we could observe no growth in the wells filled with treated CFS and inoculated with spores of *T. tonsurans*. On the contrary, profuse growth could be observed in the well containing pH-neutralized CFS of LpMYSN7 and in the control (Figure 11). This indicates that the antifungal metabolites in the CFS are acidic in nature, which prompted further investigation of CFS for its organic acid content.

### Organic acid profiling

During LC-MS/MS analysis of the CFS-LpMYSN7, a mixture of organic acids was detected (Table 4). They were identified as succinic acid, lactic acid, pyruvic acid, malonic acid, maleic acid, fumaric acid, malic acid, shikimic acid, citric acid, and hydroxycitric acid. Among these, the predominant acids were succinic and lactic acid at a concentration of 9,793.60 and 2,077.86 μg/ml, respectively.

**Table 4:** Organic acid profiling of the cell-free supernatant of *Lp*MYSN7.

**Organic acids**	**IUPAC name**	**Concentration (μg/ml)**
Lactic acid	2-Hydroxypropanoic acid	2077.86
Pyruvic acid	2-oxopropanoic acid	201.66
Malonic acid	propanedioic acid	93.96
Maleic acid	(2*Z*)-But-2-enedioic acid	11.06
Fumaric acid	(2*E*)-But-2-enedioic acid	9.67
Succinic acid	Butane-1,4-dioic acid	9793.60
Malic acid	2-Hydroxybutanedioic acid	11.66
Tartaric acid	2,3-Dihydroxybutanedioic acid	3.60
Shikimic acid	3*R*,4*S*,5*R*)-3,4,5-Trihydroxy cyclohex-1-ene-1-carboxylic acid	10.27
Citric acid	2-Hydroxypropane-1,2,3-tricarboxylic acid	11.72
Hydroxycitric acid	1,2-Dihydroxypropane-1,2,3-tricarboxylic acid	15.07

### SEM analysis of LpMYSN7 effect on *T. tonsurans*

**Figure 12:**
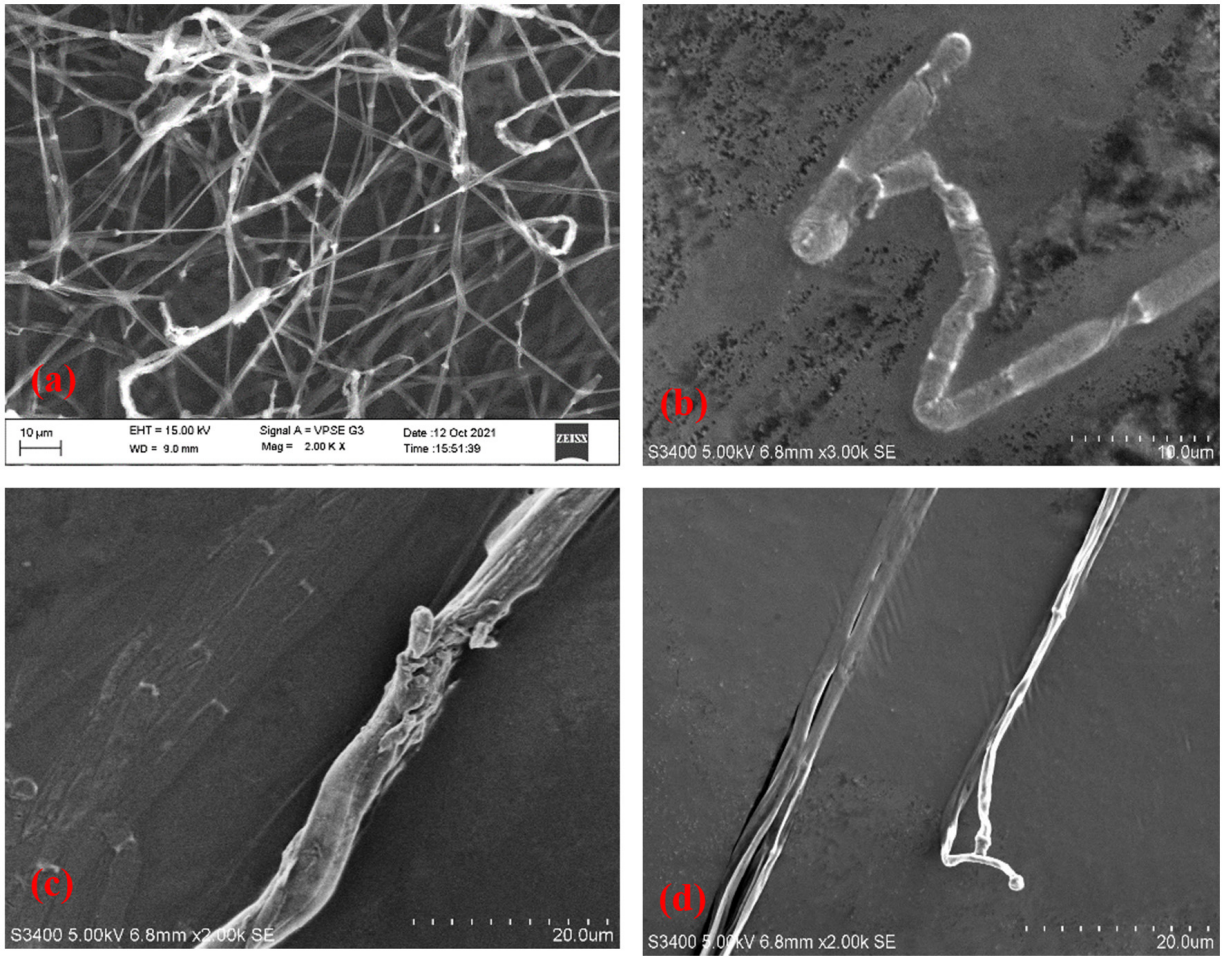
Scanning electron micrographs of *T. tonsurans*. **(a)** Control—profusely branched hyphae. **(b–d)** Scanty branching and twisting of the hyphae, disruption in the cell wall, and a bulged terminus.

Antifungal compounds present in the CFS of LpMYSN7 had an observable effect on the hyphal morphology as can be observed in the SEM images (Figure 12). Evident mycelial disintegrations were observed in the hyphal wall taken from the inhibited zone (Figure 12c), and the extent of hyphal branching was also significantly retarded (Figure 12d) when compared to the control. Mycelia were less healthy with constrictions and bulged termini (Figures 12b, d), whereas, in the case of the control, no such effects could be detected. The mycelia were intact and healthy (Figure 12a).

## Discussion

This study focussed on the screening of antifungal activity of *Lactiplantibacillus* spp. isolated from the fermented rice beverage, Haria. Previously, the same ethnic food was investigated for the composition of microbial consortia that developed during fermentation. It was found that molds and yeasts were present in high numbers during the early fermentation period followed by *Lactobacillus* and *Bifidobacteria*, which were predominant at the end (Ghosh et al., 2015). Ray et al. (2017) further investigated the probiotic attributes of *Bifidobacterium* sp MKK4 isolated from Haria and suggested its use as a potent probiotic agent. In our study, we have made an attempt to isolate and study the probiotic characteristics of *Lactiplantibacillus* spp. from this beverage and also studied the antifungal activity of one of the strains LpMYSN7 against the dermatophytic fungi *Trichophyton tonsurans*, an emerging pathogen of tinea capitis (Müller et al., 2021).

Of the 20 LAB strains that were isolated, the MYSN7 strain later identified as *L. plantarum* exhibited maximum antifungal activity during the preliminary screening. This strain was selected to further evaluate its probiotic and anti-*Trichophyton* activity. The purpose of doing such a study is to explore the application of a probiotic isolate with potential antifungal activity that could be used against skin infections. LAB have long been proven to have different types of antimicrobial activity and are regarded safe owing to their presence in fermented foods (Rawat et al., 2018) and their association with the mammalian intestine (Reale et al., 2015). Studies have convincingly shown that the intake of probiotics helps to stop and treat skin conditions (Fabbrocini et al., 2016). Some early studies recommend the advantages of probiotics, e.g. that they introduce healthy microorganisms to the gut and make a barrier to prevent inflammation, which may trigger certain skin conditions (Holz et al., 2017). The World Health Organization has researched the result of probiotics on skin diseases and stated that there is strong proof of the role of probiotics in treating skin diseases. Thus, the development of probiotic formulations that could be applied topically is an exciting area of analysis, and also many makers are currently exploring the idea of adding either cells or extracts of probiotics in skin care products such as moisturizers, lotions, cleansers, and peels (Bernard and Fr, 2017; Angeles and Angeles, 2019).

*L. plantarum* MYSN7 strain, which was identified by 16S rRNA sequencing, has indicated potential probiotic characteristics with an acid tolerance of 75% and 70% survival percentage in pH 3 and pH 2, respectively. This acid tolerance character of *L. plantarum* was attributed to the transcriptional and physiological response of the species under acid stress. There is an upregulation of gene expression involved in the production of alkali and synthesis of saturated fatty acids and cyclopropane fatty acids of cell membranes in acid-challenged cells, causing a significant reduction in membrane fluidity (Huang et al., 2016). Our study displayed the bile salt tolerance level of LpMYSN7 as 68.73% in 0.3% ox gall after 3 h of incubation. This is a moderately bile-tolerant strain as shown by Hamon et al. (2011) who compared the bile salt tolerance properties of nine *L. plantarum* strains. The investigation led to the identification of six proteins that may play a key role in the bile salt response and adaptation in *L. plantarum*: two glutathione reductase enzymes involved in protection against oxidative stress caused by bile salts, a cyclopropane fatty acyl-phospholipid synthase protein implicated in maintaining cell envelope integrity, a bile salt hydrolase enzyme, an ABC transporter, and an F0F1-ATP synthase that play a part in the active removal of bile-related stress factors (Argyri et al., 2013). The study further proves that this tolerance character is strain-dependent as some of the strains of *L. plantarum* were highly resistant to bile, showing a survival percentage of 85%−97%, while some strains had a moderate tolerance of 66 to 81%. Hence, the study of bile tolerance needs to be carried out for any new isolate.

Cell surface hydrophobicity is an important property of the adhesion capacity of probiotics to the intestinal mucosa. Microbial adhesion to hydrocarbons (MATH) analysis of *L. plantarum* strains by Yadav et al. (2013) indicated that while 40% of the strains have a low percentage of hydrophobicity (< 25%), only 20% have a higher percentage of hydrophobicity (>35%). The strain *Lp91* has exhibited the maximum hydrophobicity of 37.9 and 39.4% to toluene and hexadecane, respectively. All these were fecal isolates. In another study, 18 strains of *L. plantarum* isolated from various sources were analyzed for their adhesion capacity to xylene and chloroform. The five strains from infant feces show extraordinarily high hydrophobicity, ranging from 65% to 80%, while the remaining strains from traditional fermented fish and shrimp intestines had hydrophobicity ranging from 30% to 50%. This indicates that the adhesion capacity of the strains depends on their isolation habitats (Buntin et al., 2017). Comparatively, our strain LpMYSN7 isolated from fermented rice beverage has shown strong cell surface hydrophobicity of 48.87% and an auto-aggregation percentage of 80.62%. Furthermore, the organism has exhibited effective antibacterial activity against common human pathogens, such as *S. aureus, S. paratyphi, K. pneumoniae, E. coli*, and *P*. *aeruginosa*. There are several mechanisms through which this biological activity can be accomplished, the principal of which include competition for nutrients and adhesion sites, inducing changes in environmental conditions that are unfavorable to pathogenic bacterial growth, production of antimicrobial metabolites, and finally modulating the host immune responses (Nair et al., 2017). Comparative analysis of the antimicrobial activity of different probiotic strains has shown that *L. plantarum* often displays a broad capacity to inhibit pathogens among *Lactobacilli* species (Davoodabadi et al., 2015; Divyashree et al., 2021, 2022).

In addition, knowing about the isolate's susceptibility to commonly used antibiotics is essential to find the risk of transfer of antibiotic resistance genes (Argyri et al., 2013). LpMYSN7 has shown sensitivity to most of the commonly used antibiotics. This sensitivity pattern gives assurance that the organism is totally safe as a probiotic agent. Though the isolate shows resistance to vancomycin, it is detected to be an intrinsic resistance character (Delcour et al., 1999). In addition, mutations in chromosomes leading to drug resistance have been detected in lactobacilli (Taylor et al., 2015). However, the risk of transfer is considered very low in both intrinsic resistance and acquired resistance due to chromosomal mutations (Gueimonde et al., 2013).

The anti-*T. tonsurans* activity of LpMYSN7 was tested in both solid and liquid media. In the agar overlay assay, the isolate showed strong inhibition of +++ (inhibition zone covering 8–10% of the agar plate) when compared to the other two strains selected for the study. This prompted us to select this strain to proceed with further analysis. When the chosen strain was tested against the fungi in a liquid medium (modified MRS broth), the result was encouraging as the antifungal activity was found to be comparable to that of the agar overlay assay. During the 14-day incubation period, the growth of *T. tonsurans* in the presence and absence of LpMYSN7 was measured by fungal biomass gain. The biomass of the fungi did not show any increase at the end of the incubation period with LpMYSN7 cells when compared to the control in which the weight of the mycelial mat increased exponentially from 0.201 to 5.720 g after 14 days. In a similar study by Guo et al. (2012), five LAB strains, namely *Lactobacillus reuteri R2, Lactobacillus reuteri ee1p, Lactobacillus brevis JJ2P, Lactobacillus arizonensis R13*, and *Lactobacillus casei R20*, which had shown strong antifungal activity during preliminary screening, were tested against *T. tonsurans*, and among those, *L. reuteri* R2 was found to be the most active against the fungi, affirming that the antifungal activity of the LAB is strain specific.

To see the antifungal activity of the cell-free supernatant of LpMYSN7 (CFS-LpMysN7), different concentrations of the sterile CFS were added to the medium inoculated with *T. tonsurans* spores. At a minimum of 6% concentration, the fungal growth was inhibited almost fully with negligible biomass of the fungi (0.125 ± 0.03 g) when compared to the control, which gave 1.632 ± 0.3 g after the same period of incubation. Such biomass assays were usually not performed with dermatophytic fungi, and for the first time, we tested the effect of LAB cells and their CFS on the growth of such fungi by studying the effect on biomass. However, the biomass assay has been performed for studying the control of other fungi, such as *Aspergillus parasiticus, F. proliferatum*, and *Aspergillus ochraceus* (Rao et al., 2019; Deepthi et al., 2016; Adithi et al., 2022).

The germination of the microconidia of *T. tonsurans* and mycelial growth was examined in the presence of CFS and compared with the control. At 6% concentration, the percentage of conidial germination was between 4 and 6% even after 72 h of incubation. However, at the same incubation period, 100% germination was seen with the control. This is comparable with another study carried out by Guo et al. (2012) in which it was detected that in the control, maximum germination (95%) of *T. tonsurans* microconidia was obtained after the incubation period of 21 h and in the presence of 10% freeze-dried CFS of the *L. reuteri* producer strain (cfsp), the germination and mycelial growth were completely inhibited. Interestingly, we could obtain the same effect with our crude extract.

A study of the MIC of freeze-dried CFS powder (CFSp-LpMYSN7) was carried out, which showed visual inhibition of the fungi in the microtiter plate at a concentration of 8 mg/ml with a conidial germination inhibition of approximately 90%, whereas the standard antifungal drug showed the same level of inhibition at 1.0 μg/ml with a conidial inhibition percentage of 92.9%. This difference in concentration could be because of the crude nature of our CFS powder when compared to the highly purified standard antifungal drug. To know the exact nature of the antifungal compound that is the crude extract, CFS characterization was performed, and from this, it was inferred that the antifungal effect is mainly because of the acidic nature of the extract. In organic acid profiling, the predominant acids found included succinic acid (9,793.60 μg/ml) and lactic acid (2,077.86 μg/ml). Previously, *L. plantarum* has been shown to produce organic acids, such as phenyl lactic acid (Maikhan and Researcher, 2018), benzene acetic acid, 2-propenyl ester (Wang et al., 2012), oleic acid (Rao et al., 2019), 10-octadecenoic acid, palmitic acid, heptadecanoic acid, stearic acid, and lauric acid (Deepthi et al., 2016). Nevertheless, the antifungal effect seemed to be maximal with the use of a mixture of organic acids rather than any single acid (Danial et al., 2021).

In addition to this, we wanted to see the effect of LpMYSN7 on the morphology of *T. tonsurans*. SEM analysis of the treated fungal hyphae showed different morphological anomalies when compared to the untreated control. The same anomalies have been detected in *T. tonsurans* when it was made to grow in the presence of a plant product, i.e., chicory extracts in which the hyphae become warped, with amorphous fibrillar extruded material and twisted hyphae (Mares et al., 2005).

Future experiments will be focussed on studying the effect of LpMYSN7 cells and its CFS in *in vivo* conditions to know the prospects of using it as a potential antidermatophytic formulation.

## Conclusion

This study demonstrated that *L. plantarum* MYSN7 has been identified as a new probiotic candidate with antifungal attributes against *Trichophyton tonsurans*. The *L. plantarum* MYSN7 and its CFS were able to control *T. tonsurans* at the hyphal level, biomass production inhibition, and the inhibition of conidial germination. It will further be tested for the isolate's ability to control biofilm formation in the dermatophyte, which has been implicated as the important virulence factor in causing infection. This study highlighted the occurrence of probiotic *Lactiplantibacillus* spp. in this type of rice-based fermented beverage, and there is enough scope to exploit their bio-therapeutic potential in the future.

## Data availability statement

The datasets presented in this study can be found in online repositories. The names of the repository/repositories and accession number(s) can be found in the article/Supplementary material.

## Author contributions

PRV analyzed the data and wrote the manuscript. PRV and IP carried out the research activities. MYS, SD, and RS gave technical guidance during the experiment and assisted in software handling. MYS and PRV designed the research and edited and submitted the final version of the manuscript. All authors read and approved for publication.

## Supplementary material

Figure S1 Hemolytic activity on blood agar plate **(a)** MYSN7 showing no hemolysis **(b)** Control showing beta hemolysis.
