# Supplementary figures and images for: Antifungal activity of probiotic strain Lactiplantibacillus plantarum MYSN7 against Trichophyton tonsurans
Source: Front Microbiol. 2023 Jun 14;14:1192449. doi: 10.3389/fmicb.2023.1192449 (PMC10303898; doi:10.3389/fmicb.2023.1192449)

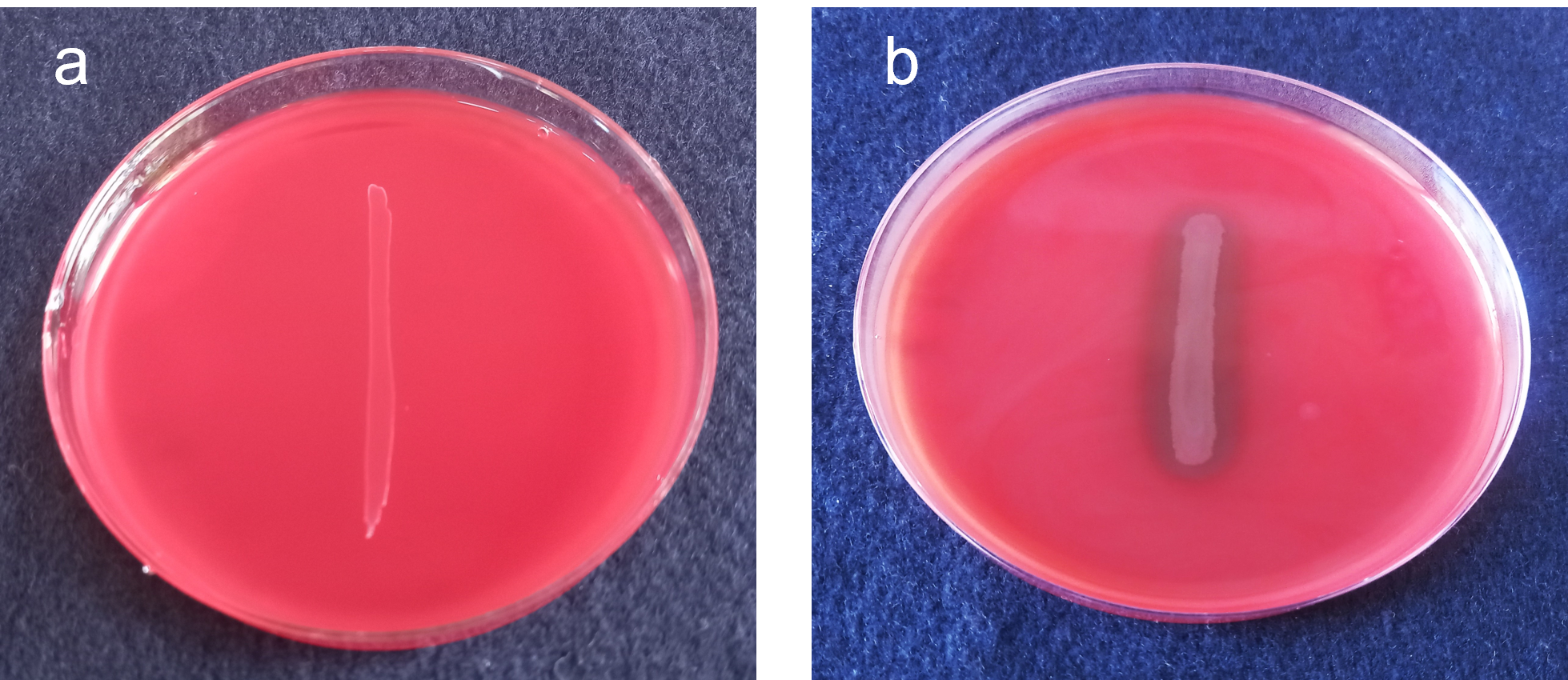

Supplement: Figure S1 — Hemolytic activity on blood agar plate (a) MYSN7 showing no hemolysis (b) Control showing beta hemolysis. [file Image_1.TIF]
